# Identification of an optimized 2′-*O*-methylated trinucleotide RNA motif inhibiting Toll-like receptors 7 and 8

**DOI:** 10.1261/rna.061952.117

**Published:** 2017-09

**Authors:** Felix C.F. Schmitt, Isabel Freund, Markus A. Weigand, Mark Helm, Alexander H. Dalpke, Tatjana Eigenbrod

**Affiliations:** 1 Department of Anesthesiology, Heidelberg University Hospital, 69120 Heidelberg, Germany; 2 Department of Infectious Diseases, Medical Microbiology and Hygiene, Heidelberg University Hospital, 69120 Heidelberg, Germany; 3 German Center for Infection Research (DZIF), Partner Site Heidelberg, 69120 Heidelberg, Germany; 4 Institute of Pharmacy and Biochemistry, Johannes Gutenberg-University Mainz, 55128 Mainz, Germany

**Keywords:** immune stimulation, TLR7, TLR8, inhibitory RNA, RNA modification, 2′-*O*-methylation

## Abstract

Bacterial RNA serves an important function as activator of the innate immune system. In humans bacterial RNA is sensed by the endosomal receptors TLR7 and TLR8. Differences in the posttranscriptional modification profile of prokaryotic when compared with eukaryotic RNA allow innate immune cells to discriminate between “host” and “foreign” RNA. Ribose 2′-*O*-methylation is of particular importance and has been reported to antagonize TLR7/8 activation. Yet, the exact sequence context in which 2′-*O*-methylation has to occur to mediate its inhibitory activity remains largely undefined. On the basis of a naturally occurring 2′-*O*-methylated RNA sequence, we performed a systematic permutation of the methylated nucleotide as well as adjacent bases and hereby identify two minimal trinucleotide motifs within a 9-mer oligoribonucleotide that are necessary and sufficient to antagonize TLR7 and TLR8 activation, respectively. Given the growing interest in the development of inhibitors of nucleic acid-sensing TLRs for therapeutic purposes, these results will facilitate the rational design of such antagonists in the future.

## INTRODUCTION

The innate immune system constitutes the first line of defense against invading pathogens by recognizing highly conserved pathogen-associated molecular patterns (PAMPs) through a limited set of germline encoded pattern recognition receptors (PRRs). RNA of both bacterial and viral origin has been identified as an activator of innate immunity that is sensed by a variety of cytosolic and endosomal receptors (Alexopoulou et al. 2001; Hemmi et al. 2002; Diebold et al. 2004; Heil et al. 2004; Lund et al. 2004; Hornung et al. 2006; Kato et al. 2008; Mancuso et al. 2009; Deshmukh et al. 2011). Importantly, different immune cells vary considerably in terms of nucleic acid receptor expression and in the profile of inflammatory mediators released. Human plasmacytoid dendritic cells (pDCs) sense bacterial RNA (bRNA) via endosomal TLR7 and produce large amounts of IFN-α (Eberle et al. 2009; Gehrig et al. 2012; Jockel et al. 2012; Eigenbrod and Dalpke 2015) while human monocytes recognize microbial RNA in a TLR8-dependent manner, triggering the production of proinflammatory cytokines including TNF and IFN-β (Cervantes et al. 2013; Bergstrøm et al. 2015; Eigenbrod et al. 2015; Krüger et al. 2015). Due to these cell-type specific TLR expression and cytokine secretion profiles, bacterial RNA-induced TNF production within PBMCs is commonly used as a read-out for TLR8-dependent monocyte activation, whereas IFN-α release within PBMCs serves as a marker for TLR7-dependent pDC activation.

As bacterial and host RNA are composed of the same basic building blocks, the ability of innate immune cells to discriminate between “self” and “nonself” RNA is an essential, yet challenging task. In this regard, differential modification profiles of eukaryotic when compared with prokaryotic RNA have been proposed to be of decisive importance (Koski et al. 2004; Karikó et al. 2005; Karikó and Weissman 2007). To date, more than 100 RNA modifications have been identified that can either be incorporated at the nucleobase or at the ribose (Cantara et al. 2011; Machnicka et al. 2013). Host and bacterial RNA not only differ in the abundance of modified nucleotides, that is, eukaryotic RNA is in general more extensively modified than its bacterial counterpart, but also in the kind of modifications incorporated (Cantara et al. 2011; Motorin and Helm 2011; Machnicka et al. 2013). Of note, it could be demonstrated that the immunostimulatory capacity of different purified RNA subtypes inversely correlated with the number of modified nucleotides (Karikó et al. 2005).

Several studies identified 2′-*O*-methylation of the ribose, a modification which is more abundant in eukaryotic RNA, as a suppressor of TLR7 and TLR8 activation in human pDCs and monocytes, respectively (Robbins et al. 2007; Sioud et al. 2007; Hamm et al. 2010; Vaishnaw et al. 2010; Gehrig et al. 2012; Jockel et al. 2012; Kaiser et al. 2014; Rimbach et al. 2015). Yet, RNA 2′-*O*-methylations can also be found in certain bacteria and might be misused as an immune escape mechanism (David 2012; Gehrig et al. 2012; Jockel et al. 2012). Indeed, a single 2′-*O*-methylation of guanosine at position 18 (Gm18) within the D-loop in selected *Escherichia coli* tRNA isoacceptors including *E. coli* tRNA^Tyr^ not only rendered these specific tRNAs nonstimulatory but also acted as TLR7 and TLR8 antagonist. Thus, Gm18 tRNA suppressed immune activation by otherwise stimulatory RNA (Gehrig et al. 2012; Jockel et al. 2012; Rimbach et al. 2015). In the case of TLR7, the antagonistic effect of 2′-*O*-methylated RNA has been proposed to result from competition with stimulatory RNA for receptor binding (Hamm et al. 2010; Rimbach et al. 2015). Subsequent studies were able to identify a [DmR] motif (D = all but C; R = purine) as a functional determinant underlying the lack of TLR7 stimulation by 2′-*O*-methylated tRNA (Kaiser et al. 2014). Yet, it remains unclear if the DmR motif mediates only the nonstimulatory or also the antagonistic effect on TLR7 activation and if the bases adjacent to the dinucleotide motif are also relevant for the observed effect. Likewise, a detailed analysis of the sequence constraints underlying TLR8 antagonism is pending. Thus, in the present study we set out to characterize and optimize a common minimal sequence motif that is necessary and sufficient to inhibit both TLR7 and TLR8. Inhibitory oligoribonucleotides (ORNs) might be used for therapeutic purposes in diseases associated with aberrant immune responses toward “self” RNA, for example, the autoimmune disease systemic lupus erythematosus.

## RESULTS AND DISCUSSION

### 2′-*O*-methylation of all nucleotides except cytidine at position 18 efficiently attenuates TLR7 and TLR8 activation

**FIGURE 1.**
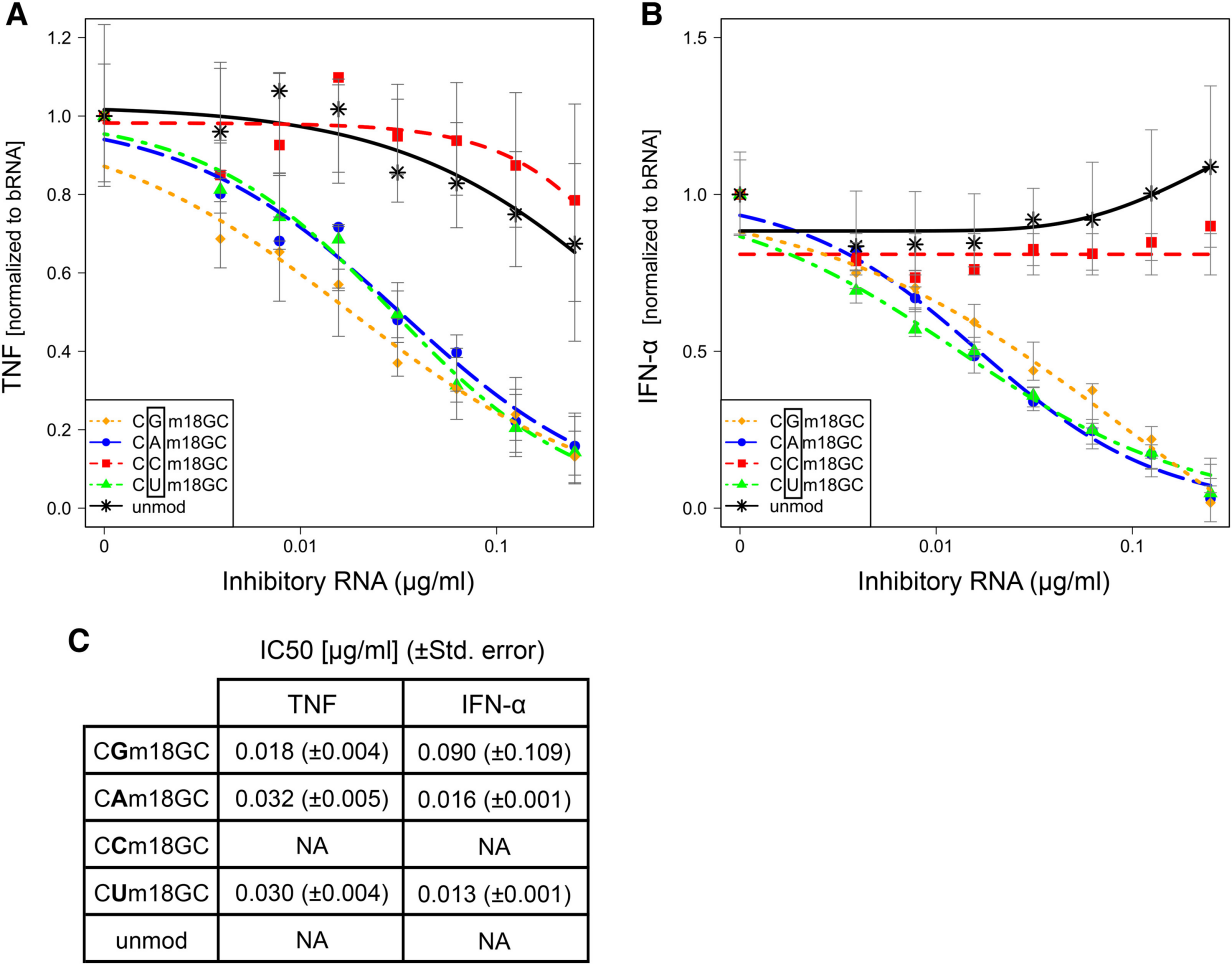
Effect of base permutation at position 18 in *E. coli* tRNA^Tyr^ on TLR7 and TLR8 activation by bacterial RNA. Human PBMCs were cotransfected overnight with 0.5 µg/mL bacterial RNA and different concentrations of the indicated 2′-*O*-methylated or unmethylated control ORN derived from the 5′ sequence of *E. coli* tRNA^Tyr^ (concentrations of inhibitory RNA were 0.25, 0.125, 0.0625, 0.031, 0.015, 0.0078, and 0.0039 µg/mL). The nucleotide at position 18 was permutated as indicated. The motif 5′-CGm18GC-3′ corresponds to the sequence found in native *E. coli* tRNA^Tyr^. Levels of (*A*) TNF and (*B*) IFN-α were measured in cell-free supernatants by ELISA. Data were normalized to cytokine production induced by bacterial RNA alone to account for donor variation. Each data point represents the mean value of five different donors, except for the unmodified ORN (*n* = 4) and the two lowest concentrations of inhibitory RNA for TNF (*n* = 3). Curve fit and IC__50__ (µg/mL) (*C*) were calculated with R software as described in Materials and Methods. The error bars represent the confidence interval of the model.

A short tRNA fragment derived from *E. coli* tRNA^Tyr^ bearing a 2′-*O*-methylation at position G18 (Gm18) has previously been demonstrated to inhibit TLR7- and TLR8-mediated immunostimulation by a variety of stimulatory RNA species as efficiently as the respective full-length tRNA (Rimbach et al. 2015). We therefore made use of this 26-mer oligoribonucleotide (ORN) to further characterize the sequence constraints that are necessary and sufficient for dominant-negative silencing of TLR7/8 activation in human PBMCs. To this end, immune stimulation of PBMCs by bacterial RNA was tested in the presence of ORNs with different sequences. In an initial approach, a permutation of the 2′-*O*-methylated nucleotide at position 18 (nomenclature corresponding to the native *E. coli* tRNA^Tyr^ sequence) was performed and the inhibitory capacity of the resulting ORN was evaluated. Titration of the tRNA fragments to a constant concentration of bacterial RNA was performed to allow for a better discrimination of differences in the immunosuppressive potential of the tested ORNs and to permit the calculation of IC_50_ values. An unmodified ORN served as specificity control. Activation of TLR8 in monocytes was determined by analyzing the secretion of TNF, whereas IFN-α release served as a read-out for TLR7-dependent pDC activation (Eberle et al. 2009; Gehrig et al. 2012; Jockel et al. 2012; Eigenbrod et al. 2015; Krüger et al. 2015). Notably, methylation of all bases except for cytidine resulted in an efficient silencing of both TLR7 and TLR8 (Fig. 1A–C). The detrimental effect of a 2′-*O*-methylated cytidine on dominant-negative immunosilencing is in line with the notion that Cm18-modified full-length *E. coli* tRNA^Tyr^ remained largely stimulatory in a previous study (Kaiser et al. 2014). Similarly, 2′-*O*-methylcytidine failed to attenuate IFN-α induction by siRNA even when the abundance of modified residues exceeded 25% of all nucleotides (Eberle et al. 2008).

### A purine base following the 2′-*O*-methylated nucleotide is essential for TLR8 silencing

**FIGURE 2.**
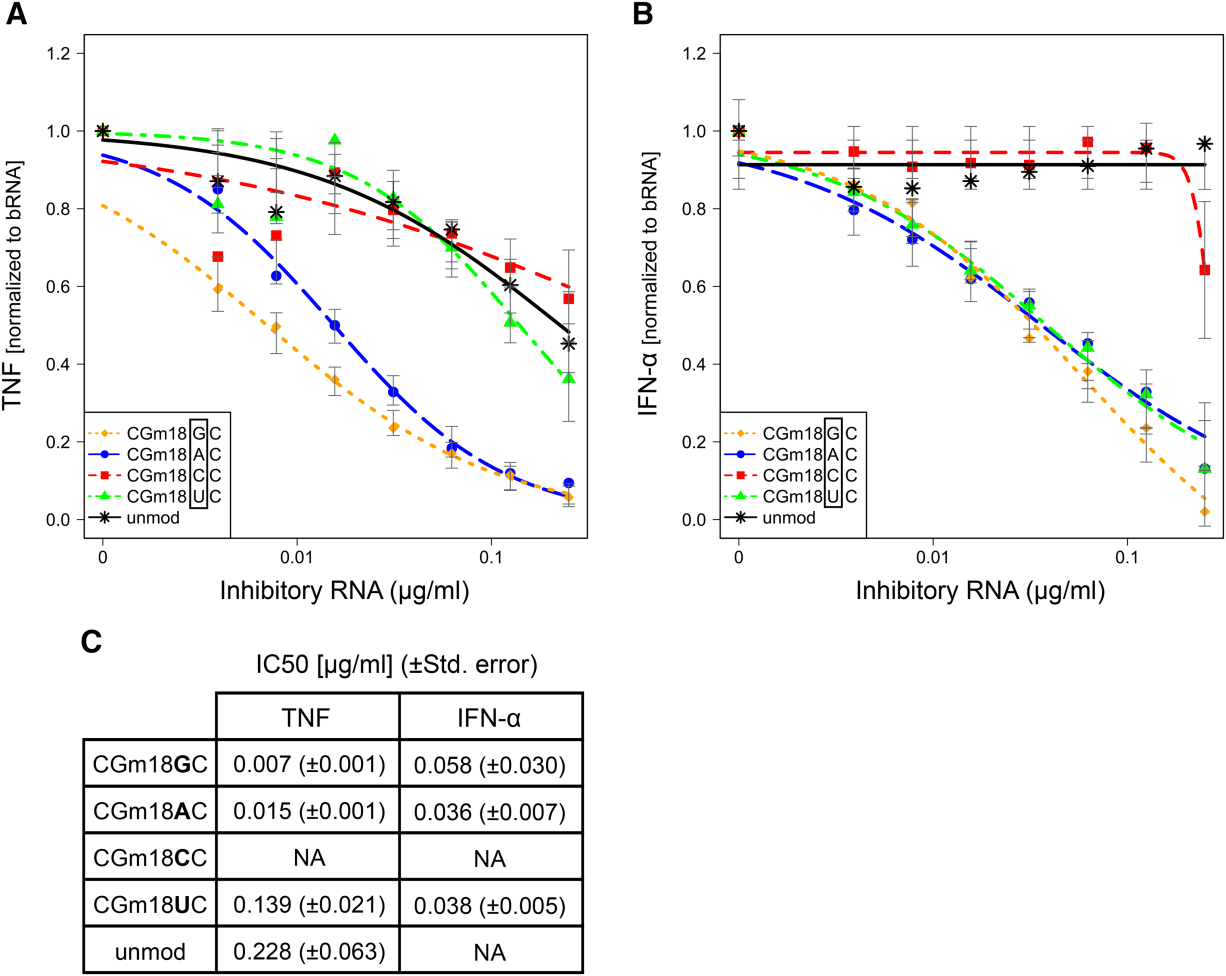
Effect of base permutation at position 19 in *E. coli* tRNA^Tyr^ on TLR7 and TLR8 activation by bacterial RNA. Human PBMCs were stimulated and analyzed for secretion of (*A*) TNF and (*B*) IFN-α as described in Figure 1. Curve fit and IC__50__ (µg/mL) (C) were calculated with R software. The base at position 19 was permutated as indicated. For TNF, each data point represents the average value of seven to eight different donors, except for the two lowest concentrations of inhibitory RNA (*n* = 3). For IFN-α, each data point represents the average value of five different donors.

In order to investigate the influence of the bases directly adjacent to the methylated ribose, we next permutated the nucleotides at positions 17 and 19. Base permutations at position 17 demonstrated that this position was not decisive for the inhibitory capacity of the tRNA fragment as all nucleotides showed similar inhibition of both TNF and IFN-α (data not shown). In contrast, strong differences were observed for position 19, that is, one base downstream from the methylated nucleotide. Remarkably, a purine base at position 19 (Gm18G or Gm18A), as present in the native *E. coli* tRNA^Tyr^ sequence, efficiently abrogated bacterial RNA-induced TNF secretion from human PBMCs, whereas incorporation of a pyrimidine (Gm18U, Gm18C) did not impair TLR8 activation when compared with an unmodified control ORN (Fig. 2A,C). In contrast, all bases except cytidine (Gm18G, Gm18A, Gm18U motifs) antagonized TLR7-dependent IFN-α production with a similar IC_50_ (Fig. 2B,C). Together, these results unravel differences in the sequence constraints at position 19 required for TLR8 when compared with TLR7 silencing and indicate that at least a dinucleotide motif is crucial for mediating the observed antagonistic effects.

### A trinucleotide motif containing a cytidine at position 20 is most efficient in antagonizing TLR8 activation

**FIGURE 3.**
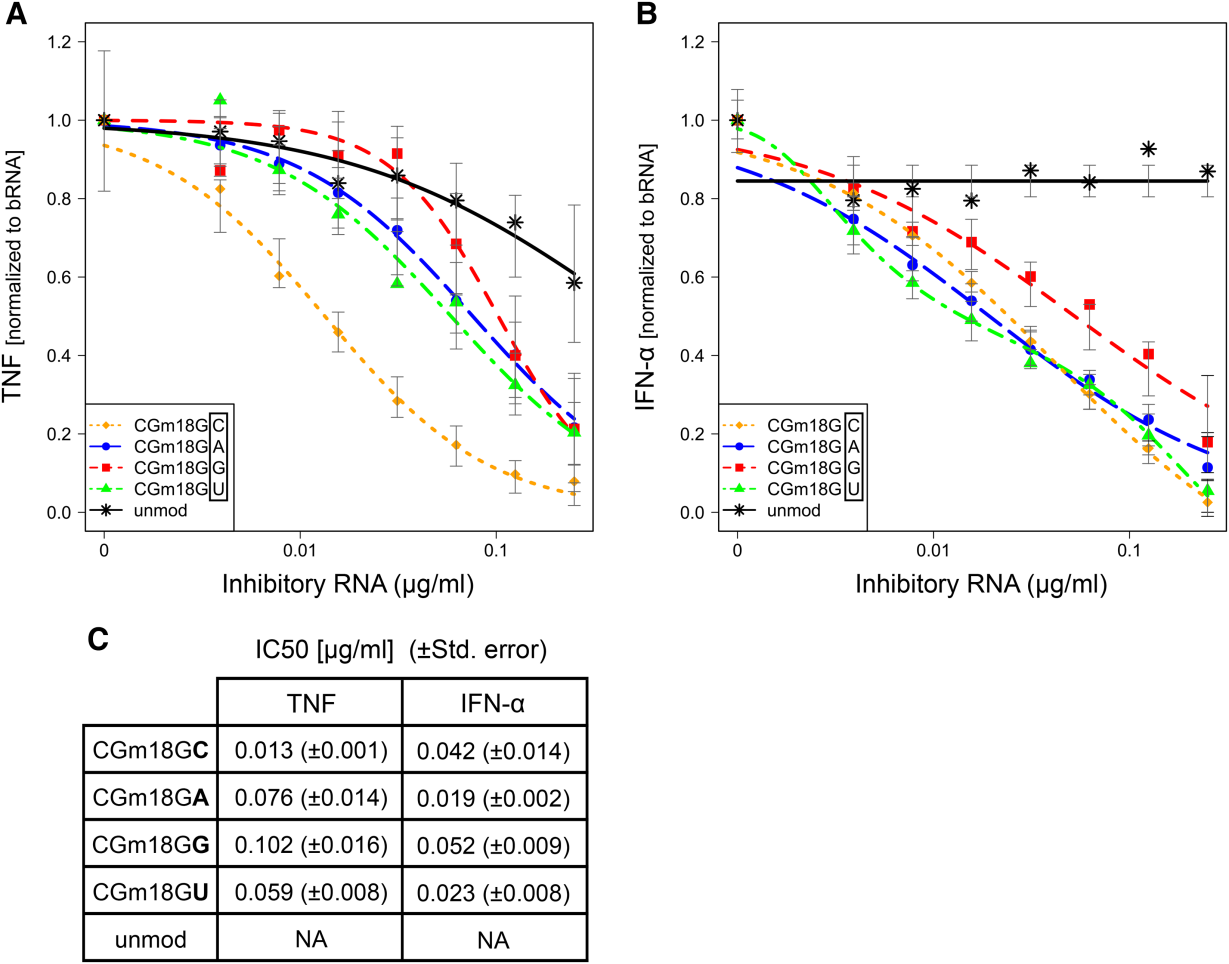
Effect of base permutation at position 20 in *E. coli* tRNA^Tyr^ on TLR7 and TLR8 activation by bacterial RNA. Human PBMCs were stimulated and analyzed for secretion of (*A*) TNF and (*B*) IFN-α as described in Figure 1. Curve fit and IC__50__ (µg/mL) (*C*) were calculated with R software. The base at position 20 was permutated as indicated. Each data point represents the mean value of five to seven different donors.

To further elucidate whether the dinucleotide motif identified so far was sufficient for immunosilencing, we next permutated the nucleotide at position 20, that is, two bases 3′ of the methylated ribose. Unexpectedly, the base at position 20 appeared to be the most discriminative one in terms of TLR8 inhibition. Indeed, the most prominent antagonistic effect on bacterial RNA-induced TLR8 activation was observed when a cytidine was incorporated (Gm18GC), as present in the native *E. coli* tRNA^Tyr^ sequence. Insertion of other nucleotides (Gm18GG, Gm18GA, Gm18GU) showed minor inhibition of TNF secretion (Fig. 3A,C). In contrast, incorporation of adenosine, uridine, or cytidine at position 20 showed equal efficiency on TLR7 silencing, whereas guanosine (Gm18GG) was slightly less efficient in inhibiting IFN-α secretion (Fig. 3B,C). Together, these results suggest that a trinucleotide motif is essential for mediating dominant negative effects on both TLR7 and TLR8 activation. For TLR8, this inhibitory sequence can be defined as [*D*m*R*C] motif (*D* = all but C [G is optimal]; *R* = G, A) and for TLR7 as [*D*m*DN*] motif (*D* = all but C; *N* = all).

Previous studies indicated that 2′-*O*-methylated RNA inhibits TLR7 activation by competing with stimulatory RNA for receptor binding (Hamm et al. 2010; Rimbach et al. 2015). It is thus likely that a similar mechanism applies for inhibition of TLR8. Interestingly, two recently published studies on the crystal structure of ligand-bound TLR7 and TLR8 revealed that both receptors possess two distinct binding sites for RNA: While TLR7 binds a guanosine at site 1 and a U-rich ssRNA at site 2, TLR8 harbors a uridine and a short UG-containing ORN at the first and second site, respectively (Geyer et al. 2015; Tanji et al. 2015; Maeda and Akira 2016; Zhang et al. 2016). Whether Dm-modified inhibitory RNA binds to either site 1 or site 2, thereby hindering access of stimulatory RNA to its receptor, remains to be determined. Yet, the low IC_50_ values required for the IFN-α and the TNF inhibitory effect suggest that Dm-modified RNA displays a higher binding affinity to the receptor than unmodified stimulatory RNA.

### Length constraints and positional effects of the Dm motif on TLR8 inhibition

**FIGURE 4.**
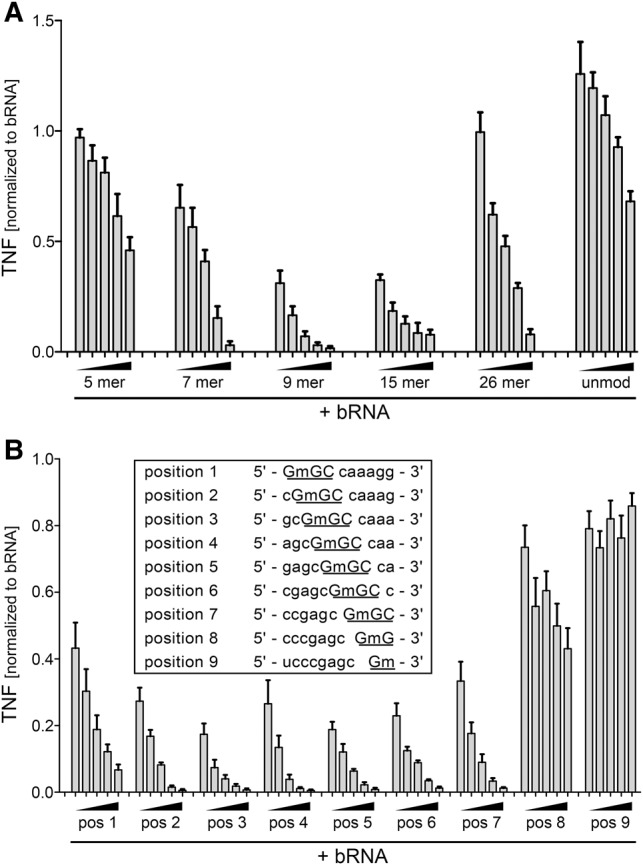
Analysis of length constraints and positional effects of the Gm motif on TLR8 inhibition. Human PBMCs were transfected overnight with 0.5 µg/mL bacterial RNA in the presence of different concentrations of (*A*) 2′-*O*-methylated tRNA fragments of the indicated length or (*B*) a 9-mer tRNA fragment with permutated positioning of the Gm motif as indicated. (*A*,*B*) Wedges indicate concentrations of inhibitory RNA of 0.25, 0.125, 0.0625, 0.031, and 0.015 µg/mL. Levels of TNF were measured in cell-free supernatants by ELISA. All values were normalized to cytokine production induced by bacterial RNA alone to account for donor variation. Data represent mean values (±SEM) of 2–3 (*A*) or 3 (*B*) different donors.

As all previous experiments in this study were performed using a 26-mer tRNA fragment, we next asked the question which minimal length was required for immunosilencing. To this end, the tRNA fragment was shortened at both the 5′ and 3′ end to obtain ORNs of variable length featuring a central 2′-*O*-methylated guanosine. Interestingly, a length of nine bases seemed to provide the most beneficial effects on TLR8 suppression, whereas the effect was lost when the ORN was shortened to 5 nt (Fig. 4A). Given that the TLR8 antagonistic effect of 2′-*O*-methylated RNA is likely mediated by competition for receptor binding, it is conceivable that beyond the inhibitory motif additional effects such as hydrophobic interactions or hydrogen bonds might strengthen the interaction of the inhibitory ORN with the receptor.

To further investigate the importance of the exact positioning of the Gm motif within a defined ORN, we varied the localization of the 2′-*O*-methylated guanosine within the 9-mer ORN. Hereby, with a terminal placement of the methylation, the inhibitory trinucleotide motif was partially disrupted. Localization of Gm at positions 2–7 inhibited bacterial RNA-induced cytokine secretion from human PBMCs with similar efficiencies, while a slight decrease in the inhibitory potential was observed upon incorporation of Gm at the 5′ terminus (position 1). Of note, placement of Gm toward the 3′ end, that is, at positions 8 or 9, was detrimental to immunosilencing of both TNF and IFN-α, in line with the notion that the inhibitory trinucleotide motif “Gm18GC” was compromised under those conditions (Fig. 4B and data not shown for IFN-α). Together, these results demonstrate that the exact position of the inhibitory motif within the ORN is less important as long as the complete trinucleotide motif is preserved.

In summary, we identify in the current study two trinucleotide motifs that potently antagonize TLR7 and TLR8 stimulation by RNA. Although a 2′-*O*-methylated nucleotide is required, our results suggest that the 2′-*O*-methylation is only functional within a trinucleotide motif whose optimal sequence slightly differs for TLR7 and TLR8 inhibition. Indeed, the sequence motif for TLR8 [*D*m*R*C] appears to be more stringent than that identified for TLR7 [*D*m*DN*], especially regarding the two bases directly downstream from the methylated ribose. Remarkably, the strongest attenuation of both TLR7 and TLR8 activation was observed for the “GmGC” motif, which corresponds to the sequence context present in native *E. coli* tRNA^Tyr^. While the motif “Gm18G19” is highly conserved within tRNA isoacceptors of both prokaryotic and eukaryotic origin, position 20 can be occupied by either a cytidine or—even more frequently—by a dihydrouridine (Cantara et al. 2011; Machnicka et al. 2013). Given the striking influence of position 20 on immunosilencing regarding TLR8, the inhibitory potential of such a GmGD (D = dihydrouridine) motif remains to be determined.

The differences in the sequence constraints required for TLR7 when compared with TLR8 silencing identified in the present study might also explain the findings of a recent investigation by Jung et al. (2015). The authors described that a 2′-*O*-methylation within a synthetic 18S rRNA-derived RNA sequence prevented IFN-α secretion without affecting production of IL-6 in human PBMCs and therefore concluded that this specific 2′-*O*-ribose methylation converted a TLR7/TLR8 agonist into a TLR8-specific ligand. Of note, the 2′-*O*-methylation in their study appeared in a “GmGU” context, which according to our current data, is predicted to impair TLR7 but not TLR8-mediated immune responses. Yet, the two studies are not entirely comparable as we were focusing on TLR antagonistic properties of 2′-*O*-methylated ORNs while Jung et al. (2015) evaluated the mere presence or absence of immunostimulation.

Over the past few years it became evident that certain autoimmune diseases such as systemic lupus erythematosus or psoriasis are associated with an inappropriate activation of endosomal TLRs by endogenous RNA and DNA with subsequent release of proinflammatory mediators. Thus, the development of inhibitors targeting nucleic acid-sensing TLRs has gained increasing interest and the respective compounds are currently under investigation in preclinical and clinical trials (Suárez-Fariñas et al. 2013; Zhu et al. 2013; Celhar and Fairhurst 2014; Wu et al. 2015). The identification and characterization of a sequence motif that potently attenuates TLR7/8-induced immune responses as presented in the current study might therefore facilitate the rational development of TLR antagonists for therapeutic applications in the future.

## MATERIALS AND METHODS

### Reagents

RPMI 1640 containing stable glutamine was purchased from Biochrom, FCS was obtained from Gibco, Ficoll (1.078 g/mL) from Pan Biotech, TRIzol from Thermo Fisher Scientific, R848 and CpG 2216 from InvivoGen, and DOTAP (*N*-[1-(2, 3-dioleoyloxy)propyl]-*N*, *N*, *N*-trimethylammonium methylsulfate) from Roth.

### Oligoribonucleotides used in the study

RNA sequences used for stimulation experiments were: 2′-*O*-methylated tRNA fragment corresponding to the native *E. coli* tRNA^Tyr^ sequence: 5′-GU GGG GUU CCC GAG CGmG CCA AAG GGA-3′; unmodified control tRNA fragment: 5′-GU GGG GUU CCC GAG CGG CCA AAG GGA-3′. In some experiments, the methylated base (underlined) or the preceding or following nucleobases (shown in boldface) (corresponding to positions 18, **17**, **19**, and **20** in native *E. coli* tRNA^Tyr^) were permutated within the framework of the 26-mer as follows:

Position 17: **A**GmGC; **G**GmGC; **U**GmGC. Position 18: C**Am**GC; C**Cm**GC; C**Um**GC. Position 19: CGm**A**C; CGm**C**C; CGm**U**C. Position 20: CGmG**A**; CGmG**G**; CGmG**U.**

9-mer Gm18: 5′-GAG CGmG CCA-3′; 7-mer Gm18: 5′-AGC GmGC C-3′; 5-mer Gm18: 5′-GCGm GC-3′. All oligoribonucleotides were custom synthesized from Biomers.

### Preparation of total bacterial RNA

*Staphylococcus aureus* (ATCC 25923) was grown in Luria-Bertani (LB) medium (Merck) and harvested within the mid-log phase growth. After a digestion step with lysozyme (c = 40 mg/mL, 20 min at 37°C), total bacterial RNA was isolated using TRIzol reagent according to the manufacturer's protocol. The obtained RNA underwent a further purification step using the RNeasy mini kit (QIAGEN), including an on-column DNA digestion step. Purity of the RNA preparations was validated by determining the 260/230 nm and 260/280 nm extinction ratio by NanoDrop (Thermo Scientific).

### Isolation and stimulation of human immune cells

Human peripheral blood mononuclear cells (PBMCs) were isolated from heparinized blood of healthy volunteers upon informed consent and approval by the local ethical committee by standard Ficoll-Hypaque density gradient centrifugation (Ficoll 1.078 g/mL). PBMCs were resuspended in RPMI 1640 supplemented with 10% heat-inactivated FCS. For transfection experiments, bacterial RNA was encapsulated with DOTAP at a ratio of 3 µL DOTAP per 1 µg of RNA in serum-free medium according to the manufacturer's protocol. Unless otherwise indicated, cells were stimulated with bacterial RNA at a final concentration of 500 ng/mL. Where indicated, 2′-*O*-methylated inhibitory RNA or an unmodified control RNA were mixed with bacterial RNA at different ratios prior to encapsulation with DOTAP. Reverse transfection was performed at a density of 4 × 10^5^ cells/well (PBMCs) in a 96-well flat bottom plate. Cells were incubated overnight in a humidified 5% CO_2_ atmosphere at 37°C. As positive control, PBMCs were stimulated with TLR7/8-agonist R848 (1 μg/mL). For cytokine measurement, levels of TNF (BD) and IFN-α (eBioscience) were determined in cell-free supernatants by ELISA. All values were normalized to cytokine production induced by bacterial RNA alone to account for donor variation. For stimulation with bacterial RNA alone, cytokine levels ranged approximately between 6000 and 15,000 pg/mL for TNF and approximately between 250 and 750 pg/mL for IFN-α.

### Statistical analysis

Curve fit and IC_50_ values for inhibitory RNA were calculated as follows: We first evaluated which dose response model best fit the data. Four functions were tested with variations in the numbers of parameters: log-logistic function (2–5 parameter), log-normal dose response function (2–4 parameters), Weibull function (2–4 parameters), and the fractional polynomial-logistic dose–response function. The best fit was selected based on the following criteria: the log likelihood value, Akaike's information criterion (AIC), the estimated residual standard error and the *P*-value from a lack-of-fit test. Most of the responses fit well to a log-logistic function (with 2–4 parameters). The only exceptions were the expression of IFN-α for the CGm18GU and unmodified motif (best model: fractional polynomial-logistic dose–response functions). The error bars indicated in Figures 1–3 represent the confidence interval of the model. All analyses were performed with R software version 3.3.0 and the package drc (R Core Team 2013; Ritz et al. 2015).
